# The Virome of Cocoa Fermentation-Associated Microorganisms

**DOI:** 10.3390/v16081226

**Published:** 2024-07-31

**Authors:** João Pedro Nunes Santos, Gabriel Victor Pina Rodrigues, Lucas Yago Melo Ferreira, Gabriel Pereira Monteiro, Paula Luize Camargo Fonseca, Ícaro Santos Lopes, Brenno Santos Florêncio, Aijalon Brito da Silva Junior, Paulo Eduardo Ambrósio, Carlos Priminho Pirovani, Eric Roberto Guimarães Rocha Aguiar

**Affiliations:** 1 Department of Health Sciences, Universidade Estadual de Santa Cruz, Ilhéus 45662-900, BA, Brazil; jpnsantos.bio@uesc.br; 2 Department of Biological Science, Center of Biotechnology and Genetics, Universidade Estadual de Santa Cruz, Ilhéus 45662-900, BA, Brazil; gvprodrigues.ppggbm@uesc.br (G.V.P.R.); lucasmelobiomed@gmail.com (L.Y.M.F.); pirovani@uesc.br (C.P.P.); 3 Department of Biochemistry and Immunology, Universidade Federal de Minas Gerais, Belo Horizonte 31270-901, MG, Brazil; 4 Department of Genetics, Institute of Biological Sciences, Universidade Federal de Minas Gerais, Belo Horizonte 31270-901, MG, Brazil; 5 Department of Engineering and Computing, Universidade Estadual de Santa Cruz, Ilhéus 45662-900, BA, Brazil; brennosantosflorencio@gmail.com (B.S.F.); aijalonjunior@gmail.com (A.B.d.S.J.); peambrosio@uesc.br (P.E.A.)

**Keywords:** cocoa fermentation, virome, microorganisms, metatranscriptome, *Lenarviricota*, *Narnaviridae*

## Abstract

*Theobroma cacao* plantations are of significant economic importance worldwide, primarily for chocolate production. During the harvest and processing of cocoa beans, they are subjected to fermentation either by microorganisms present in the environment (spontaneous fermentation) or the addition of starter cultures, with different strains directly contributing distinct flavor and color characteristics to the beans. In addition to fungi and bacteria, viruses are ubiquitous and can affect the quality of the fermentation process by infecting fermenting organisms, destabilizing microbial diversity, and consequently affecting fermentation quality. Therefore, in this study, we explored publicly available metatranscriptomic libraries of cocoa bean fermentation in Limon Province, Costa Rica, looking for viruses associated with fermenting microorganisms. Libraries were derived from the same sample at different time points: 7, 20, and 68 h of fermentation, corresponding to yeast- and lactic acid bacteria-driven phases. Using a comprehensive pipeline, we identified 68 viral sequences that could be assigned to 62 new viral species and 6 known viruses distributed among at least nine families, with particular abundance of elements from the *Lenarviricota* phylum. Interestingly, 44 of these sequences were specifically associated with ssRNA phages (*Fiersviridae*) and mostly fungi-infecting viral families (*Botourmiaviridae*, *Narnaviridae*, and *Mitoviridae*). Of note, viruses from those families show a complex evolutionary relationship, transitioning from infecting bacteria to infecting fungi. We also identified 10 and 3 viruses classified within the *Totiviridae* and *Nodaviridae* families, respectively. The quantification of the virus-derived RNAs shows a general pattern of decline, similar to the dynamic profile of some microorganism genera during the fermentation process. Unexpectedly, we identified narnavirus-related elements that showed similarity to segmented viral species. By exploring the molecular characteristics of these viral sequences and applying Hidden Markov Models, we were capable of associating these additional segments with a specific taxon. In summary, our study elucidates the complex virome associated with the microbial consortia engaged in cocoa bean fermentation that could contribute to organism/strain selection, altering metabolite production and, consequently, affecting the sensory characteristics of cocoa beans.

## 1. Introduction

The chocolate manufacturing process is deeply intertwined with the process of fermenting cocoa beans. After harvesting, the cocoa fruit goes through several essential stages that are decisive for the development of important sensory characteristics for the production of high-quality chocolate, such as the complexity of flavor, texture, and aroma, highly valued by manufacturers and consumers of chocolate [1]. The sensorial value of chocolate increases mainly during the fermentation of cocoa beans. This process is fundamental to achieving an excellent-quality product. During this phase, compounds such as organic and volatile acids emerge that intensify the desired flavors and aromas in the final product [2,3].

To better understand the cocoa fermentation process, it is essential to explore the biological agents involved. A diversity of microorganisms, including yeasts and lactic acid bacteria, catalyze important biochemical transformations, creating flavor precursors that define the sensory profile of fermented cocoa [4]. The interaction of these microorganisms is fundamental in determining the final sensory properties, such as flavor and aroma. Understanding and managing this diversity is crucial to optimizing production [5]. Therefore, microorganisms in cocoa pulp affect the dynamics of metabolites produced in the fermentation process, directly impacting the aroma and quality of the product. Variations in microbial load can affect the types and concentrations of metabolites, which can lead to significant variations in the chemical compounds present, potentially impacting the final product [6,7]. In this diverse microbiota, viruses can also be found, as has been observed in wine production [7,8]

Viruses also play a significant role in fermentation, influencing the evolution of bacterial communities and directly impacting the quality of fermented products [9]. The presence of viruses can both inhibit and potentiate certain metabolic processes, revealing how viruses can influence the fermentative capacity of strains [10,11]. Genes from the auxiliary metabolic pathways encoded by viruses are capable of rewiring host metabolic pathways, which can sustain, accelerate, or interrupt important metabolic processes in fermentation [11]. However, despite the importance and influence of these parasites in the fermentation process, there is a lack of information regarding their diversity and abundance, and how it correlates with microorganisms’ dynamics over time.

Here, we explored the complex virome associated with microorganisms involved in the cocoa fermentation process and its dynamics, discussing its possible effect on the fermentation process and, therefore, on the quality of the final product.

## 2. Materials and Methods

### 2.1. Retrieval of RNA Deep Sequencing Libraries

To assess the virome associated with the microorganisms involved in the cocoa fermentation process, we took advantage of three RNA deep sequencing libraries derived from samples collected at different cocoa bean fermentation time points: 7 h (ERR4077213), 20 h (ERR4077214), and 68 h (ERR4077215). Cocoa fermenting samples were derived from plantations in Limon Province, Costa Rica [12]. Detailed information about the libraries can be found in Supplementary Table S1.

### 2.2. Metaviromic Analyses

The analysis of the public RNA-seq libraries involved an extensive pipeline previously described [13]. Quality control of raw reads was assessed using FastQC (galaxy version 0.74+galaxy0) [14]. Trimmomatic (galaxy version 0.36.6) [15] was then used to remove low-quality reads (Phred < 20) and adaptor sequences. Next, the trimmed reads were aligned against the *Theobroma cacao* genome (GCF_000208745.1) using Bowtie2 (galaxy version 2.5.3+galaxy0) [16] with default settings to remove any *T. cacao*-related reads. Initially, unaligned reads were assembled using Trinity (galaxy version 2.15.1+galaxy1) [17]. Due to sample complexity, an additional integrative assembly step was performed using RNAviralSPAdes (version 3.15.5) [18], Trinity (version 2.15.1), Trans-ABySS (version 2.0.1) [19], and IDBA (version 1.1.3) [20] to obtain more reliable and complete sequences. To identify viral transcripts, a sequence similarity search was conducted on the assembled contigs using Diamond (galaxy version 2.0.15+galaxy0) [21] in BlastX mode, utilizing the NCBI Viral RefSeq database (https://ftp.ncbi.nlm.nih.gov/refseq/release/viral/ accessed on 14 March 2024) as a reference. Sequences that exhibited an identity and coverage below 90% when compared to known viruses were considered new viral species.

### 2.3. Manual Curation of Viral Genomes

Non-retroviral sequences were filtered based on a minimum size threshold of 500 nucleotides. The filtered sequences were manually examined using an online version of BLAST against the Nucleotide (NT) and Protein (NR) databases. An overview of the best hits at the nucleotide and amino acid levels for each viral sequence can be found in Supplementary Table S2. To remove redundancy, sequences were clustered using CD-HIT (Version 4.8.1) [22] with an identity and coverage threshold of 95 percent. Subsequently, reassembly was performed using CAP3 (Galaxy Version 2.0.0) [23] with default parameters to improve the assembly of specific sequences. Nucleotide-level alignment between sequences was conducted, focusing on the 3′- and 5′-end alignments, without gaps, allowing for a maximum of 2 non-adjacent mismatches within 20 nucleotides for assembly correction and/or extension. Aiming to extend the length of characterized sequences, we took the sequences not aligned to the RefSeq viral database and performed CD-HIT and CAP3. Then, we merged the aligned RefSeq viral sequences with the non-aligned sequences and again performed CD-HIT and CAP3. Finally, we manually inspected the sequences through nucleotide alignment. Graphical visualization of the alignment allowed for the identification of chimeras, which were removed. The complete set of sequences assembled and characterized in this work is available in Supplementary File S1.

The ORFfinder tool [24] was utilized to predict open reading frames (ORFs) within the sequences, adapting the genetic code and initiation codes when necessary. InterPro [25], HMMER [26], and CD-Search [27] were applied to identify conserved domains.

### 2.4. Phylogenetic Analysis

All sequences identified in this study related to polymerases or polyproteins which presented domains and a size greater than 500 nt were used to reconstruct the phylogeny and corroborate the classification based on the similarity search using the BLAST tool [28]. The viral sequences were evaluated in ORFFinder. The amino acid sequences were used to assess the presence of the RNA-dependent RNA polymerase (RdRp) domain in the HMMER program. After confirming the presence of the RdRp or related domains, the sequences were subjected to a similarity search in BLAST, and the five best hits were used to construct the dataset for phylogenetic analysis. Furthermore, viral sequences containing the RdRp domain recognized by the International Committee on Taxonomy of Viruses (ICTV—https://ictv.global/ accessed on 24 March 2024) as a reference for each genus of the families *Mitoviridae*, *Narnaviridae*, *Botourmiaviridae*, *Partitiviridae*, *Totitviridae*, and *Fiersviridae* were used to compose the dataset and help classify the identified viral sequences. The final dataset presented 546 sequences. The dataset was aligned using the MAFFT program [29]. Maximum likelihood was inferred by the IQ-TREE [30] program using 1000 replicates as bootstrap and choosing the best evolutionary model to construct the analysis. The final phylogeny was visualized by FigTree and edited manually. Detailed information about the sequences used for phylogenetic reconstruction can be found in Supplementary Table S3.

### 2.5. Abundance and Diversity Analyses

Salmon software (Galaxy Version 1.10.1+galaxy2) [31] was employed to evaluate the transcriptional activity of virus-derived sequences. A comprehensive overview of the transcriptional activity of these viral sequences is presented in Supplementary Table S4. We conducted a comprehensive assessment of alpha diversity utilizing the richness [32] index through the VEGAN package (version 6) in R [33].

### 2.6. Assessment of Molecular Characteristics of Putative Viral Sequences

The analysis of 3′-end stem–loop formation was performed using the RNAfold web service [34] with standard parameters. The alignment of the 3′ ends of each segment was conducted using T-Coffee to identify and visualize identical regions among the small sequences [35].

### 2.7. HMM Analysis

Hidden Markov Model (HMM) analysis was performed to search for RNA-dependent RNA polymerase (RdRp) domains present in the contig sequences. For this, the NeoRdRp2 database [36] was used using the HMMSCAN application, from the HMMER (3.4) package [37]. The analysis was performed on the total ORFs of the dataset under the standard genetic code.

To analyze potential segments of multi-segmented Narnavirus, three customized HMMs were created with RNA1, RNA2, and RNA3 segments from different segmented Narnavirus species previously described in the literature (Supplementary Table S5). A total of 84 amino acid sequences were used in the models with the RdRp and hypothetical protein domains. These models were used to help characterize the undefined Narnavirus segments.

## 3. Results

### 3.1. Metaviromic Analysis

To comprehend the virome dynamics during cocoa bean fermentation, we performed a comprehensive metatranscriptomics analysis of the three publicly available libraries representing time points at 7, 20, and 68 h of fermentation. These libraries collectively contained 285,082,459 raw reads, with an average of approximately 95,027,486 reads per library (Supplementary Table S1). Upon read assembly, we obtained 25,657, 18,991, and 17,021 contigs in the samples derived from 7, 20, and 68 h of fermentation, respectively. The initial assembly yielded 64 non-redundant putative viral sequences. However, utilizing the integrative assembly pipeline, we increased the number of viral sequences identified to 85, ranging from 502 to 5074, with an average of 1345 nt. Overall, the application of this enhanced approach leads to the identification of 21 new sequences ranging from 507 to 2748 nt long, showing similarity to viral elements only at the amino acid level, with 9 of them showing similarity to polymerases.

Since the sequence similarity analysis revealed multiple possible viral species, and some of them had incomplete lengths, we performed an extra step of assembly using viral hits plus no-hit contigs. This strategy allowed us to extend 14 sequences, with extension lengths ranging from 22 to 504 nucleotides. The most significant extension occurred in the sequence that exhibited an alignment with a virus from the *Fiersviridae* family, ultimately reaching 1085 nt. Sequences with similarity to viruses from the family *Mitoviridae* (four sequences) and unclassified viruses (two sequences) exhibited extensions ranging from 70 to 364 nucleotides. Two double-stranded RNA (dsRNA) virus sequences were found to be elongated. One contig related to the *Totiviridae* family exhibited an extension of 195 nucleotides. The other showed an increase of 124 nucleotides, with the best alignment to elements from the *Partitiviridae* family (Supplementary Figure S1). A single sequence, exhibiting high similarity to elements from the *Narnaviridae*, was identified as a chimera and discarded based on the ORF structure.

After manual curation, 68 non-redundant sequences longer than 500 nt were kept. Sequence similarity searches revealed that a large portion of the assembled contigs were not related to known viral species at the nucleotide level (91%), while six sequences represent species previously described (Supplementary Table S2).

Among the putative new viral sequences, 48 contained conserved domains such as RNA-dependent RNA polymerase (RdRp), capsid, and viral maturation (Supplementary Table S2 and Supplementary File S1). The remaining sequences either lack known conserved domains due to their small size or are truly novel, such as segments of narnavirus that are not well described in the literature.

**Figure 1:**
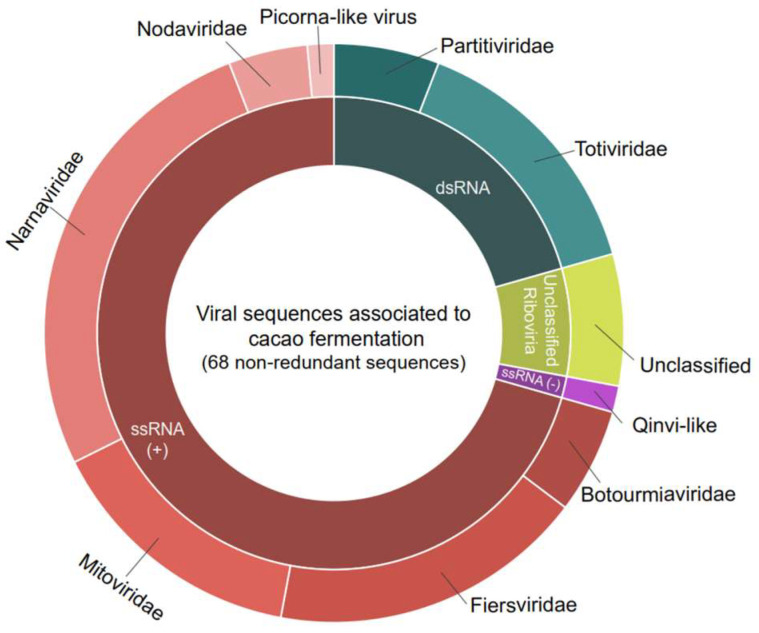
Diversity of viral sequences found in cocoa fermentation-associated microorganisms. In total, 68 sequences were found, including *Botourmiaviridae*: 4; *Totiviridae*: 10; *Narnaviridae*: 18; *Mitoviridae*: 10; *Partitiviridae*: 4; *Fiersviridae*: 12; *Nodaviridae*: 3; Picorna-like virus and Quivi-like: 1; and unclassified virus: 5.

Detailed analysis of the contigs’ best hits revealed a diverse array of viruses, encompassing both positive single-stranded RNA viruses (ssRNA+), negative single-stranded RNA viruses (ssRNA-), and double-stranded RNA viruses (dsRNA). At the family level, the ssRNA+ viruses included representatives from *Botourmiaviridae*, *Fiersviridae*, *Mitoviridae*, *Narnaviridae*, and *Nodaviridae* (Figure 1). The ssRNA- viruses included the *Qinviridae* family. The dsRNA viruses were characterized by the presence of elements related to the *Totiviridae* and *Partitiviridae* families (Figure 1).

### 3.2. Characterization of Known Viruses

Six contigs exhibited high sequence similarity to known viruses at both the nucleotide (>85% identity with >95% of coverage) and amino acid levels. Among these, two complete sequences according to its closest relatives, measuring 3124 and 1416 nucleotides, were related to the unclassified Nodaviridae sp. virus from the *Nodaviridae* family, presenting ps-ssRNAv_Nodaviridae_RdRp, Peptidase_A6 superfamily, and Noda_Vmethyltr superfamily domains commonly found in elements of this family (Supplementary Table S2 and Figure S2). The remaining four contigs with lengths of 2588, 839, 595, and 511 nucleotides likely represent partial sequences from elements within the *Narnaviridae*, *Mitoviridae*, *Picornaviridae*, and *Totiviridae* families, respectively. Indeed, they presented both family-specific domains, such as the Mitovir_RNA_pol superfamily for *Mitoviridae* and unspecific ones, such as the ps-ssRNAv_RdRp-like superfamily, which can be found in elements from the *Narnaviridae* and *Picornaviridae* families (Supplementary Table S2 and Figure S2).

### 3.3. Characterization of Novel Viruses

Sixty-two sequences were related to known viral species only at the amino acid level. They showed similarity to elements from at least eight viral families within four phyla: Lenarviricota (*Botourmiaviridae*, *Narnaviridae*, *Mitoviridae*, and *Fiersviridae*), Duplornaviricota (*Totiviridae* and *Partitiviridae*), Kitrinoviricota (*Nodaviridae*), and Negarnaviricota (*Qinviridae*), plus unclassified elements from the Riboviria realm.

#### 3.3.1. Lenarviricota

Viruses of the family *Botourmiaviridae* contain an ssRNA+ genome, which can be either mono- or multi-segmented. These genomes predominantly exist as mono-segmented with only one ORF encoding a polymerase, ranging in size from 2 to 5 kb. *Botourmiaviridae* primarily infect fungi and plants [38], and possibly oomycetes [39]. Their replication sites are concentrated in the cytoplasm; however, research has also shown the presence of these viruses in mitochondria [38].

**Figure 2:**
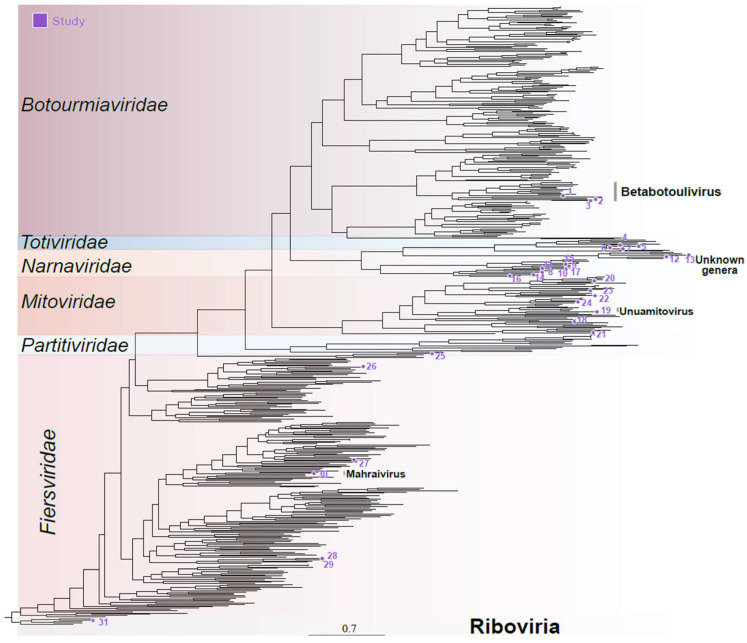
Phylogenetic analysis of cocoa fermentation-associated viruses. These sequences concern members of the families *Mitoviridae*, *Narnaviridae*, *Botourmiaviridae*, *Partitiviridae*, *Totiviridae*, and *Fiersviridae* plus one unclassified Riboviria. Numbers represent the ID of the sequence used for the phylogenetic reconstruction, detailed in Supplementary Table S2.

Four transcripts related to viruses within the *Botourmiaviridae* family were identified. These included three complete polymerases, ranging from 1692 to 2624 nucleotides in length, and one incomplete sequence of 659 nt long. These transcripts displayed sequence similarity at the amino acid level and contained the same domains as their closest hits, including the ps-ssRNAv_RdRp-like domain. The exception was the shorter transcript, which did not display any conserved domains (Supplementary Figure S2). The phylogenetic analysis utilized the three larger transcripts with the largest predicted ORFs, based on the mold, protozoan, and coelenterate mitochondrial code as well as the mycoplasma/spiroplasma genetic code. Interestingly, these transcripts contained a polymerase domain and an ORF larger than half of their nearest known hit. Concordantly with sequence similarity analysis, phylogeny indicates that the assembled sequences belong to the *Botourmiaviridae* family, specifically within the Betabotoulivirus genus (Figure 2—sequences 1, 2, and 3).

Viruses belonging to the *Narnaviridae* family are primarily non-segmented, featuring an ssRNA+ genome ranging from 2.3 to 3.6 kb in size. They encode only one polymerase protein and mostly infect fungal hosts [40]. While the genomes of most narnaviruses consist solely of an RNA-dependent RNA polymerase (RdRp) gene, certain viruses in this family possess multiple RNA segments [41]. Seventeen transcripts, ranging from 503 to 3393 nucleotides, were found to show sequence similarity at both the nucleotide level and the amino acid level to elements from the *Narnaviridae* family. Among these, nine were related to polymerase sequences, while two others were similar to hypothetical proteins potentially related to a narnavirus: RNA2 and RNA3. The remaining six transcripts did not display any conserved domains likely representing partial fragments. The polymerase sequences containing conserved RNA-directed RNA polymerase domains (Supplementary Table S2) were included in the phylogenetic analysis, which reinforced these sequences belong to the *Narnaviridae* family, although it was not possible to determine any specific genus (Figure 2—sequences 8–17).

Viruses belonging to the family *Mitoviridae* present a non-segmented ssRNA+ genome ranging from 2.1 to 4.9 kb in size. These genomes encode for an RdRp and are known to replicate within mitochondria [42]. Nine transcripts, with lengths ranging from 602 to 2547 nucleotides, were identified as closely related to elements from the Mitoviridae family. Indeed, they presented conserved domains including RNA-dependent RNA polymerase mitoviral and DNA/RNA_pol_sf (IPR043502) domains, consistent with what was observed for mitoviruses. (Figure 2 and Figure S2). Phylogenetic analysis confirmed their relationship with elements from the *Mitoviridae* family, although only one species could be classified at the genus level—Unuamitovirus (Figure 2—sequence 19).

*Fiersviridae* consists of bacteriophages characterized by an ssRNA+ genome, with sizes typically ranging from 3.4 to 4.6 kb [43]. Twelve transcripts were identified based on sequence similarity at the amino acid level, belonging to viruses within the *Fiersviridae* family, ranging from 507 to 3700 nucleotides long. These included one complete polyprotein, seven incomplete maturation proteins, and four incomplete polymerases (Supplementary Figure S2).

Conserved domains such as the ps-ssRNAv_RdRp-like superfamily, phage maturation protein, and RNA replicase beta-chain were notable, showing strong similarity to their closest related viruses (Supplementary Table S2). For the phylogenetic analysis, we selected polymerase sequences presenting conserved domains, which confirmed their assignment, and Fierviruses, with one sequence clustering with elements from the *Mahraivirus* genus (sequence 30), hereafter named Mahraivirus limonense (MVL) (Figure 2).

#### 3.3.2. Duplornaviricota

Viruses belonging to the family *Totiviridae* present a mono-segmented double-stranded RNA (dsRNA) genome ranging from 4.6 to 7 kb. They feature overlapping open reading frames (ORFs) that encode a capsid protein (CP) and an RNA-dependent RNA polymerase (RdRp). Their known hosts include fungi and protozoa [44]. Nine assembled transcripts, spanning 530 to 5087 nucleotides, presented sequence similarity at the amino acid level to unclassified elements from the *Totiviridae* family. The transcripts included four complete polyproteins and five incomplete polymerase and coat transcripts. Some of these sequences encoded proteins containing conserved domains commonly found in totiviruses, such as RNA-dir_pol_PSvirus (IPR007094), RNA-dir_pol_luteovirus This happens due to the number of analyzed sequences. It would be possible only by re-doing the whole analysis. (IPR001795), and L-A virus major coat protein superfamily domains, as well as the DNA/RNA_pol_sf (IPR043502) (Supplementary Figure S2). Only the four complete sequences and one partial sequence containing a polymerase domain were included in the phylogenetic analysis. In concordance with sequence similarity, the sequences clustered with unclassified elements from the *Totiviridae* family (Figure 2).

#### 3.3.3. Pisuviricota

Viruses of the *Partitiviridae* family are non-enveloped and possess bi-segmented dsRNA genomes ranging from 3 to 4.8 kb. The RNA1 segment encodes the RdRp, while the RNA2 segment encodes the capsid protein. These viruses primarily infect fungi and plants [45]. Four transcripts, ranging from 671 to 1638 nucleotides, including complete RNA1 and RNA2 sequences as well as incomplete RNA1 and RNA2 sequences, displayed sequence similarity at the amino acid level to polymerases and capsids related to elements from the *Partitiviridae* family. Screening for conserved domains revealed the presence of RNA-directed RNA polymerase domains on the RNA1 sequences, while the RNA2 sequences did not display conserved domains as expected for viruses from this family (Supplementary Figure S2). The polymerases were used in the phylogenetic analysis, which grouped these sequences with elements from the *Partitiviridae* family (Figure 2).

#### 3.3.4. Kitrinoviricota and Negarnaviricota

The *Nodaviridae* family consists of small, spherical, non-enveloped viruses with bipartite genomes of ssRNA+. The genome includes two segments: RNA1, which encodes protein A, an RNA-dependent RNA polymerase, and RNA2, which encodes protein α, the precursor to the capsid protein. Within this family, alphanodaviruses primarily infect insects, while betanodaviruses are known to infect fish [46,47]. A single transcript, 787 nucleotides in length, demonstrated sequence similarity at the amino acid level to the polymerase of XiangYun tombus-noda-like virus 8, an unclassified member of the *Nodaviridae* family. Although it is not a complete polymerase, analysis for the presence of conserved domains revealed the presence of RdRp of positive-sense ssRNA viruses, viral RNA-dependent RNA polymerase, and DNA/RNA polymerase domains, similar to its closest hit (Supplementary Figure S2). Phylogenetic analysis was not performed for this transcript due to its short length.

The remaining six transcripts, ranging from 518 to 1415 nucleotides in length, included one smaller incomplete transcript that presented amino acid-level similarity to elements from the *Quinviridae* family, a family of negative-sense RNA viruses with genomes of 7.3–8.2 kb that have been associated with crustaceans, insects, gastropods, and nematodes [48]. The other five incomplete transcripts showed similarity to unclassified Riboviria viruses. Domain analysis revealed the absence of conserved domains in the *Quinviridae*-like transcript and two of the Riboviria sequences (Supplementary Figure S2). Consequently, these sequences were not included in the phylogenetic analysis.

All the sequences submitted to phylogenetic analysis were named according to their taxonomic designation (Supplementary Table S2).

### 3.4. Diversity and Co-Occurrence

**Figure 3:**
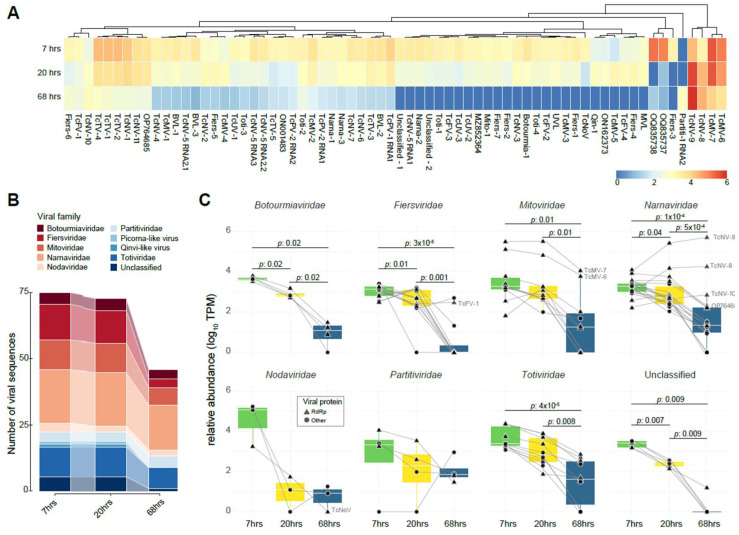
Transcriptional profile of cocoa fermentation-associated viruses along different time points. (**A**) The heatmap represents the transcriptional activity of identified viral sequences associated with cocoa fermentation. The color spectrum reflects transcription levels, ranging from high (red) to low (blue) abundance. Row clustering based on Pearson correlation group sequences with similar profiles. Abundance was normalized by transcripts per million (TPM) and plotted on a log10 scale. The conditions represented include samples from distinct time points of fermentation: 7 h, 20 h, and 68 h. (**B**) The illuvial plot displays the total number of viral sequences per viral family at distinct fermentation time points (7, 20, and 68 h). (**C**) A boxplot illustrates the TPM abundance of viral families in libraries from distinct time points of cocoa fermentation, including 7 h, 20 h, and 68 h. The Y-axis presents the normalized TPM abundance, while the X-axis distinguishes between the treatments. Statistical analysis was performed using the Wilcoxon test.

Following the virome characterization, we took advantage of the existence of public RNA libraries from distinct time points in the cocoa fermentation process to assess the influence of fermentation on the composition and dynamics of the cocoa-associated virome and vice versa. We observed that all examined viral sequences, except for the sequence Partiti-1 RNA2 (best hit with coat protein from *Saccharomyces cerevisiae partitivirus 1*), were present in at least two distinct libraries (Figure 3A). Additionally, Unuamitovirus limonense (UVL) out of 68 viral sequences exhibited higher abundance during the initial 7 and 20 h of fermentation, followed by a decline at the 68 h time point. This suggests a notable association between fermentation progress and viral abundance, mirroring the impact of fermentation on the cocoa microbiome as reported in the original paper [12] (Figure 3A). Interestingly, sequences from Theobroma cacao fermentation-associated mitovirus 6 and 7 (TcMV-6 and TcMV-7), Theobroma cacao fermentation-associated narnavirus 8 and 9 (TcNV-8 and TcNV-9), consistently showed an abundance 10–20 times higher than most other viral sequences across all libraries (Figure 3A). In the early fermentation stage libraries, a higher number of viral sequences were detected, a tendency that was reflected in the virome alpha diversity with 2.4455, 1.4783, and 0.3048 for the 7, 20, and 68 h libraries, respectively (Figure 3B). *Narnaviridae* was the most prominent viral family. In contrast, *Fiersviridae* showed the most significant decline in viral sequences in the 68 h libraries, contributing to the decrease in the family diversity (Figure 3B and Supplementary Table S4).

Analyzing the abundance per viral family, we found that all viral families, with the exception of *Nodaviridae* and *Partitiviridae*, showed a significant decline in viral abundance as fermentation progressed. The most impacted family was *Fiersviridae*. Interestingly, two *Mitoviridae* sequences showed little decrease in abundance at the final fermentation stages. Conversely, three Narnavirus sequences exhibited an increase in their abundance, nearly doubling by the end of the fermentation process as depicted in Figure 3C. Among the identified known viruses, a similar decline was observed in the percentage profile of reads per genus of distinct resident microorganisms, particularly oomycetes and yeasts, such as Phytophthora and Hanseniaspora, respectively. Among them, one of the known viruses is the Saccharomyces cerevisiae YJM1199 virus N1199, a narnavirus, which displayed a curve similar to the yeast from the genus *Hanseniaspora* (Supplementary Figure S3).

### 3.5. Characterization of Multi-Segmented Narnavirus

Our metaviromic analysis detected several narnavirus sequences; while most of them were related to uni-segmented species, one set of sequences presented high similarity at the amino acid level to a multi-segmented species. Segmented viruses of the *Narnaviridae* family have been described to contain one segment with an RdRp domain and other segments without any conserved motifs, with up to four segments [49,50]. Indeed, some of the sequences detected (Theobroma cacao fermentation-associated narnavirus 5 RNA 2.1 and 2.2, and the TcNV-5 RNA 3) had amino acid-level similarity to hypothetical protein sequences of known segmented narnaviruses with coverage greater than 80% and identity between 35% and 58%, as well as a lack of conserved domains (Plasmopara viticola lesion-associated narnavirus 4, Botrytis cinerea binarnavirus 3 and Plasmopara viticola lesion-associated narnavirus 2, respectively).

**Figure 4:**
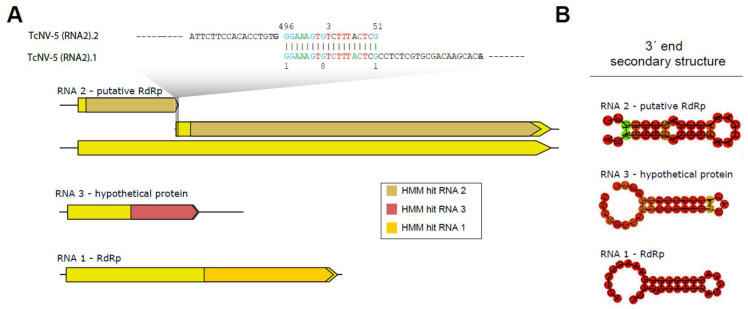
Characterization of the segmented narnavirus. A description of the TcNV-6 segments is provided, along with the hits obtained by HMM and their locations based on alignment with the trained model. The segments of the segmented narnavirus were trained as follows: RNA2 is related to putative polymerases, RNA3 is specific for hypothetical protein, and RNA1 is for polymerases (**A**). The secondary structure formed at the 3′ end of each segment (**B**).

Multi-segmented narnaviruses were only recently identified, and there is little information on molecular signatures that could help us with the characterization of these segments. Therefore, we took advantage of already characterized multi-segmented complete narnaviral sequences present on the NCBI nucleotide database to construct HMMs for each of the segments (Supplementary Table S5). Using this strategy, we identified significant hits for RNA1 (TcNV-5 RNA 1), RNA2 (TcNV-5 RNA 2.1 and 2.2), and RNA 3 (TcNV-5 RNA 3) (Supplementary Table S5). Interestingly, the two sequences displaying similarity to RNA 2 showed a smaller size than expected for the segment, raising the hypothesis that they could represent fragments of the same sequence. Indeed, manual inspection revealed the sequences share a perfect pairing of 18 nt at opposite extremities (3′ end of TcNV-5 RNA 2.2 and 5′ end of TcNV-5 RNA 2.1) (Figure 4A). The merging of these sequences produced a new sequence of 2163 nt in length with an open reading frame of 2055 nt, consistent with what was expected based on the closest related viral sequence (Figure 4A). Accordingly, RNA coverage indicated continuous coverage in the region of the merge, indicating they indeed constitute one continuous segment (Supplementary Figure S4).

Regarding the multiple segments of narnaviruses, it is known that RNA1 and RNA2 exhibit greater conservation at their termini compared to Narnavirus RNA3 [51], which might account for the observed sequence divergence. The HMM also detected similarities between TcNV-5 RNA 3 and other Narnavirus segments. The isolated segments of Narnavirus exhibit certain conserved regions, which may indicate a linkage between the sequences. Furthermore, the potential formation of secondary structures in the 3′-end region of each sequence can be observed in Figure 4B, with a pattern found in some ambigrammatic Narnaviruses [52].

## 4. Discussion

The present study aimed to investigate the dynamics of the cocoa bean fermentation virome through comprehensive metatranscriptomic analysis of three time points: 7, 20, and 68 h of fermentation. The selection of these time points was motivated by their representation of key stages in the cocoa fermentation process, as well as the requirement that the samples contain RNA of the highest integrity [12].

The cocoa fermentation process includes a complex interaction of microbiota and different substrates. Thus, the process is initiated by yeasts, followed by lactic acid bacteria and acetic acid bacteria [53]. After the initial reaction, yeasts metabolize sugars through alcoholic fermentation, producing ethanol and carbon dioxide, which contributes to the breakdown of sugars and generates an exothermic reaction, increasing the system’s temperature. This phase is also characterized by the breakdown of citric acid in the pulp by yeasts, leading to an increase in pH and the proliferation of bacteria [54].

As fermentation progressed, lactic acid bacteria (LAB) became more prevalent, contributing to the metabolic activities in the second stage of cocoa fermentation [55]. Acetic acid bacteria (AAB), such as *Acetobacter pasteurianus*, also stand out in the later stages of fermentation, contributing to the production of acetic acid and other compounds that modify acidity [56].

This multifaceted environment, the search approach allowed for the identification of viral segments rarely reported in the literature and nine polymerase sequences, which were not found in the initial assembly. These distinctions suggest that integrative assembly is advantageous for studying libraries with diverse and complex genetic material. Furthermore, the limited understanding of these viruses makes alignment-based strategy against reference sequences not the best method for identifying potential viral sequences. Consequently, the incorporation of these contigs enabled the extension of viruses, particularly those with the best alignment to families within the phylum *Lenarviricota*. This approach is of paramount importance for the exploration and adequate characterization of these less-studied viruses. Subsequently, the sequences were subjected to manual curation, resulting in the final assembly of the sequences.

Among the five known viral sequences, we identified a 2588 bp contig presenting the conserved domain of the ps-ssRNAv_RdRp-like superfamily, showing similarity to *Saccharomyces cerevisiae YJM1199 virus N1199* (2589 nt). When achieving high levels, this virus showed deleterious effects on the *Saccharomyces cerevisiae* YJM1199 strain [57]. The two known nodavirus sequences, with segments of 4749 nt and 1416 nt, were likely derived from *Nodaviridae* sp. strain Cameroon/U172471/2017/2 (3065 nt and 1370 nt), which has the same conserved domains and similar lengths. This viral strain has been identified in African human plasma [58]. Due to the handling of cocoa beans from harvesting to the box fermentation, which is a possible source of contamination by other microorganisms, it is also a source of contamination by external biological sources, which may explain the abundance of viruses from the *Nodaviridae* family, with a reduction of approximately 1000 times from the initial time point and subsequent stabilization of the abundance at 20 h. An additional hypothesis concerns viruses identified in oomycetes, as observed in *Plasmopara halstedii* and the microbiota of plants, including rice crops [59,60,61]. Their low abundance may be attributed to the increase in their host population, potentially resulting from the genus *Phytophthora*. The other known viruses are fragments of *Mitoviridae* and *Picornaviridae*, with sizes of 839 nt to 595 nt, respectively. Both best hits were derived from metagenomic studies of environmental samples such as soil, water, and feces [62].

The phylum *Lenarviricota* is frequently identified in metagenomic experiments conducted in a variety of environments, including those inhabited by extremophiles [63]. It is often associated with bacteria, yeasts, and filamentous fungi [39,64]. This phylum includes the Mycoviruses. Mycoviruses are ubiquitous, associating with various fungal groups, including those derived from the phyla Ascomycota and Basidiomycota [65]. The primary fungal genera identified in the original article are from the Ascomycota phylum, particularly from the taxonomic class Saccharomycetales. At the genus level, the most prevalent genera are *Candida*, *Hanseniaspora*, *Pichia*, *Torulaspora*, and *Saccharomyces*; however, the only genera in the top 10 are *Saccharomyces* and *Hanseniaspora*.

In general, mitoviruses replicate within the mitochondria and do not have an impact on their host. Depending on the strain, they may even be beneficial, aiding in the host’s defense mechanisms [66]. Conversely, some strains of *Cryphonectria parasitica mitovirus 1* (CpMV1), which is the prototype mitovirus found in *Cryphonectria parasitica*, have demonstrated the ability to silence the antiviral RNA interference (RNAi) pathway in species of the filamentous fungus family *Cryphonectriaceae* [67]. The relatively high abundance of TcMV-6 and 7 at 7 h, without a significant decrease like the nodaviruses, could be due to several factors. If TcMV-6 and 7 mechanisms evade or suppress the host’s antiviral responses, they could maintain higher levels of transcriptional activity. An alternative hypothesis is that the host is under stress, hampering its capacity to fight viral infection. The contig analysis indicates that TcMV-2, with a length of 2208 nucleotides (nt), is closely related to viruses of the genus *Unuamitovirus*. This genus includes viruses found in other species of filamentous fungi, is known to be associated with a reduction in mycelial formation, and may prove to be a pivotal factor in fungal development. [64,66]. The observed profile of decay suggests that TcMV-2 may share characteristics with *Unuamitovirus*, which could influence the decrease in the host population in conjunction with acidity and temperature. These viruses are classified under the genus *Betabotoulivirus*, with at least ten viruses in this genus deposited in the NCBI database. The largest sequence is 3412 base pairs (bp), found in the Entoleuca ourmia-like virus 1 isolate E112-4, and the smallest is 2545 bp, found in the Plasmopara viticola lesion-associated ourmia-like virus 4 isolate DMG-A_379541. All of these are found in hosts such as filamentous fungi or oomycetes [39,68]. The viruses of the family *Botourmiaviridae*, which have been isolated from cocoa fermentation processes, exhibit a similar pattern of decay among themselves. This suggests that they infect similar hosts, possibly an oomycete or filamentous fungus. The narnavirus results of the phylogenetic analysis and the decay profile suggest that these viruses infect similar hosts that are influenced by environmental factors.

The relationship between viruses in the *Lenarviricota* phylum is the subject of much debate, including speculation about a basal lineage of ancestral phages. In addition, there is viral diversity between different segments within the same family, which evolves in conjunction with the host, among other factors [42,69,70]. During the fermentation process of cocoa, Verce et al. (2021) demonstrated the presence of phage families, specifically dsDNA viruses belonging to the *Siphoviridae* and *Myoviridae* genera. Among the phages, we identified the Fiersviruses, a family of positive-sense single-strand RNA phages. While Fiersviruses are not as well represented in genomic databases compared to other viruses, some strains play a role in the microbial ecosystem by lysis of the bacterial cell during viral replication, which can lead to a reduction in the bacterial population. This lysis is a common strategy among bacteriophages to release new viral particles and spread to other host cells [43,71]. There has been a notable advancement in the field of phage research, with the utilization of methodologies such as the Hidden Markov Chain (HMM) [43]. Therefore, it is expected that there is a greater diversity of ssRNA phages yet to be discovered during the fermentation process. Phylogenetic analysis indicates that MVL belongs to the genus *Mahraivirus*. The genus *Mahraivirus* is represented by a single sequence, derived from a metatranscriptomic study of active sludge microbial communities from municipal wastewater-treating anaerobic digesters in the USA. This sequence is currently the only one in the NCBI Genome Database.

The results show the presence of viral transcripts from the *Totiviridae* and *Partitiviridae* families during cocoa fermentation, revealing a link between dsRNA viruses and the microbial process. Nine transcripts from the *Totiviridae* family were identified, ranging from 530 to 5087 nucleotides, indicating considerable diversity in the viral population in fermentation environments. The discovery of conserved domains in RNA polymerase and capsid proteins suggests the functionality of these viruses, indicating a possible involvement in regulatory mechanisms that control the microbiota influencing fermentation [72]. An evolutionary relationship with the *Totiviridae* family of four complete polyproteins was demonstrated in the phylogenetic analysis, implying an adaptation of these viruses to cocoa-related fungi. The lack of conserved domains in a capsid transcript may indicate an alteration in the viral protein composition, suggesting that different viral isolates may play different roles due to their genetic variability [9].

Similarly, the detection of *Partitiviridae* transcripts, a family with bi-segmented dsRNA genomes, points to the existence of these viruses in plant and fungal hosts in fermentation environments. The identification of an RNA polymerase domain in RNA1 and the absence of any conserved domain in RNA2 imply distinct functional roles for these specialized viral sequences, indicating that such interactions are intricately woven, revealing the biotic complexity found in cocoa as a host for viral and microbial entities [73].

In general, the relative abundance of the sequences declines over time. As with the total number of reads, the number of viral sequences also declines, especially for *Fiersviridae*, Picorna-like, and Quin-like viruses. This is consistent with the fact that viruses depend on a host for their replication, indicating that the population of infected hosts is also declining during the fermentation process. In other words, the variation in viral loads is closely correlated with alterations in the microbiota. The sequences that exhibited a distinct profile, those that increased over time, may be associated with specific genera of their known hosts. For example, Narnaviruses are known to infect fungi and oomycetes [49,51]. In this study, the narnaviruses TcNV-8, 9, and 10 reached their maximum abundance at 68 h, which is comparable to the read percentage profile of the yeast genus *Saccharomyces* and distinct from the oomycete *Phytophthora*, which declines at 68 h. The contigs TcFV-1 and 10, which originate from the *Fiersviridae* family, a known bacterial pathogen, exhibited behavior similar to that of AAB of the genera *Acetobacter* and *Komagataeibacter*, which suggests a possible host for these viruses.

Some sequences from the *Fiersviridae* and *Mitoviridae* families also exhibited a peak at 20 h, followed by a decline at 68 h. This profile is comparable to that of Lactiplantibacillus and Limosilactobacillus. While the profile is similar to that of LAB, there is no documented evidence of mitovirus infection among the main host genera. Instead, it is known that mitoviruses replicate in the mitochondria, which proposes a host exhibiting a similar behavior profile but not within the ten-profile dynamics.

Alignment analyses of the sequences indicated that the terminal regions of TcNV5 RNA2.1 and RNA2.2 share 18 nucleotides, effectively linking them into a single sequence. Furthermore, these analyses also suggest proximity between the sequences of TcNV5 RNA1 and RNA3, and the newly combined RNA2. These sequences showed correspondence through BLASTn and BLASTx with segments of Plasmopara viticola lesion-associated Narnavirus (RNA2 and RNA3) and with the RdRp of Rhizoctonia solani Narnavirus. However, no conserved domains were observed in the analysis of their ORFs through manual curation. The analysis using HMMs indicated domain matches of TcNV5 RNA1 with the previously described first Narnavirus segments, TcNV5 RNA3 with the third segments, and the TcNV5 RNA2 sequence with the second Narna segments. These data may indicate the presence of a new Narnavirus multi-segmented species in the samples. Models suggest that the multipartition strategy employed by certain viruses may be advantageous, as the benefits in heterogeneous environments can surpass the associated costs [74]. This perspective on multi-segmented viral fragments is important given the context of the extremophile fermentation environment and coinfection of multiple viruses.

In this context, research on viral sequences in a complex environment such as cocoa fermentation demonstrated the efficacy of metatranscriptomics and the methodology proposed by Santana et al. (2023) [13] in characterizing viromes. Furthermore, the virome of cocoa fermentation-associated microorganisms was revealed to be diverse, comprehending many viral species that were neither classified at the genus nor viral family levels. This result reinforces the need for more studies related to fermentation and the development of new strategies to identify divergent viruses. Of note, additional research is necessary to further characterize the viral genomes and their abundance, and assess their real significance during fermentation, given the prevalence of different viruses across the globe. Understanding the role of these viruses during this process is crucial for achieving the optimal quality of fermented cocoa beans.

## Data Availability

The datasets analyzed during the current study are available in the European Nucleotide Archive of the European Bioinformatics Institute (ENA/EBI) under accession numbers ERR4077213, ERR4077214, and ERR4077215. The assembled sequences are currently deposited in the NCBI nucleotide database and are also available in Supplementary File S1.

## Supplementary Materials

The following supporting information can be downloaded at https://www.mdpi.com/article/10.3390/v16081226/s1. Figure S1: Genome characteristics and conserved structures of characterized viral sequences; Figure S2: A comparative analysis of the extended sequences resulting from the curation process; Figure S3: Percentage profile of reads per genus for the ten most abundant microorganisms, as reported in the article by Verce et al., 2021 [12]; Figure S4: Density of RNAs along the merged TcNV-6 RNA2 sequence; Table S1: Table summarizing essential information for all utilized libraries; Table S2: Table summarizing the outcomes of BLASTN and BLASTX alignments for viral contigs, including details such as alignment subjects, percentage identity, and E-values; Table S3: Table summarizing all viral species included in phylogenetic analysis; Table S4: Table presenting the transcripts per million (TPM) values for viral contigs, viral alpha diversity for viral family, and Shannon entropy for library; Table S5: Table containing all ID amino acid sequences used in HMMER to characterize RNA1, RNA 2, and RNA 3 of segmented narnaviruses; File S1: Viral sequences.
