# Supplementary material for: The Virome of Cocoa Fermentation-Associated Microorganisms
Source: Viruses. 2024 Jul 31;16(8):1226. doi: 10.3390/v16081226 (PMC11359646; doi:10.3390/v16081226)
Supplement: Supplementary file 1 [file viruses-16-01226-s001.zip › Supplementary Figures - revision.pdf]

Supporting information for:

The virome of cocoa fermentation-associated microorganisms

João Pedro Nunes Santos [1], Gabriel Victor Pina Rodrigues [2], Lucas Yago Melo Ferreira [2], Gabriel Pereira Monteiro [2], Paula Luize Camargo Fonseca [2,3], Ícaro Santos Lopes [4], Brenno Santos Florêncio [5], Aijalon Brito da Silva Junior [5], Paulo Eduardo Ambrósio [5], Carlos Priminho Pirovani [2], Eric Roberto Guimarães Rocha Aguiar [5,*]

1 Department of Health Sciences, Universidade Estadual de Santa Cruz, Ilhéus 45662-900, BA, Brazil

2 Department of Biological Science, Center of Biotechnology and Genetics, Universidade Estadual de Santa Cruz, Ilhéus 45662-900, BA, Brazil

3 Department of Biochemistry and Immunology, Universidade Federal de Minas Gerais, Belo Horizonte 31270-901, MG, Brazil

4 Department of Genetics, Institute of Biological Sciences, Universidade Federal de Minas Gerais, Belo Horizonte 31270-901, MG, Brazil*

5 Department of Engineering and Computing, Universidade Estadual de Santa Cruz, Ilhéus 45662-900, BA, Brazil

*Aguiar E.R.G.R

Email: ericgdp@gmail.com

This PDF file includes:

Figure S1 to S4

Other supporting materials for this manuscript include the following

Supplementary Tables S1 to S5

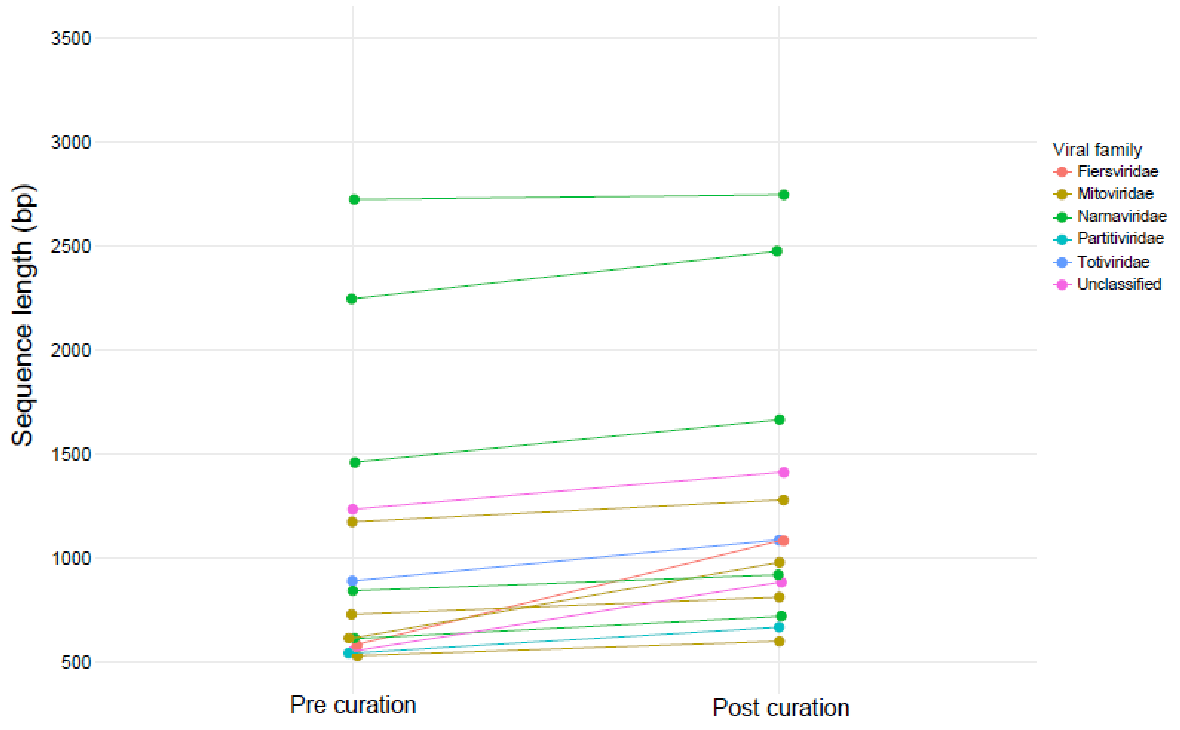

Figure S1. **Graph of elongation for 14 sequences after curation.** The figure presents the assembly of 14 sequences with contigs not aligned to reference viral proteins. Each line represents a sequence, with the inclination of the line directly proportional to the increase in sequence length after curation.

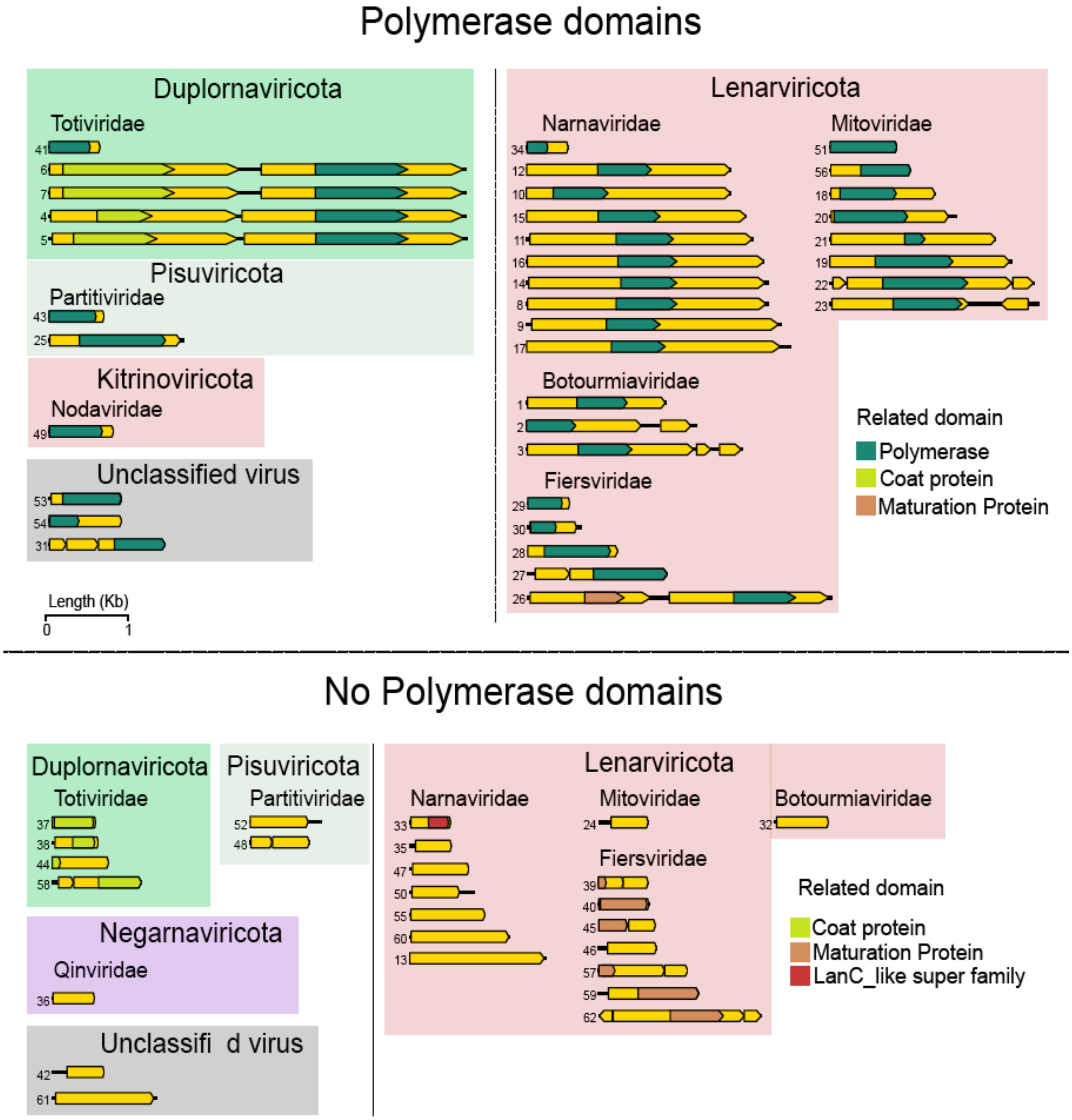

Figure S2. **Genome characteristics and conserved structures of characterized viral sequences**. Figure displays the open reading frames (ORFs) and conserved domains of characterized viral sequences. The sequences are categorized by viral order, highlighting those with RdRp conserved domains and those with other conserved domains.

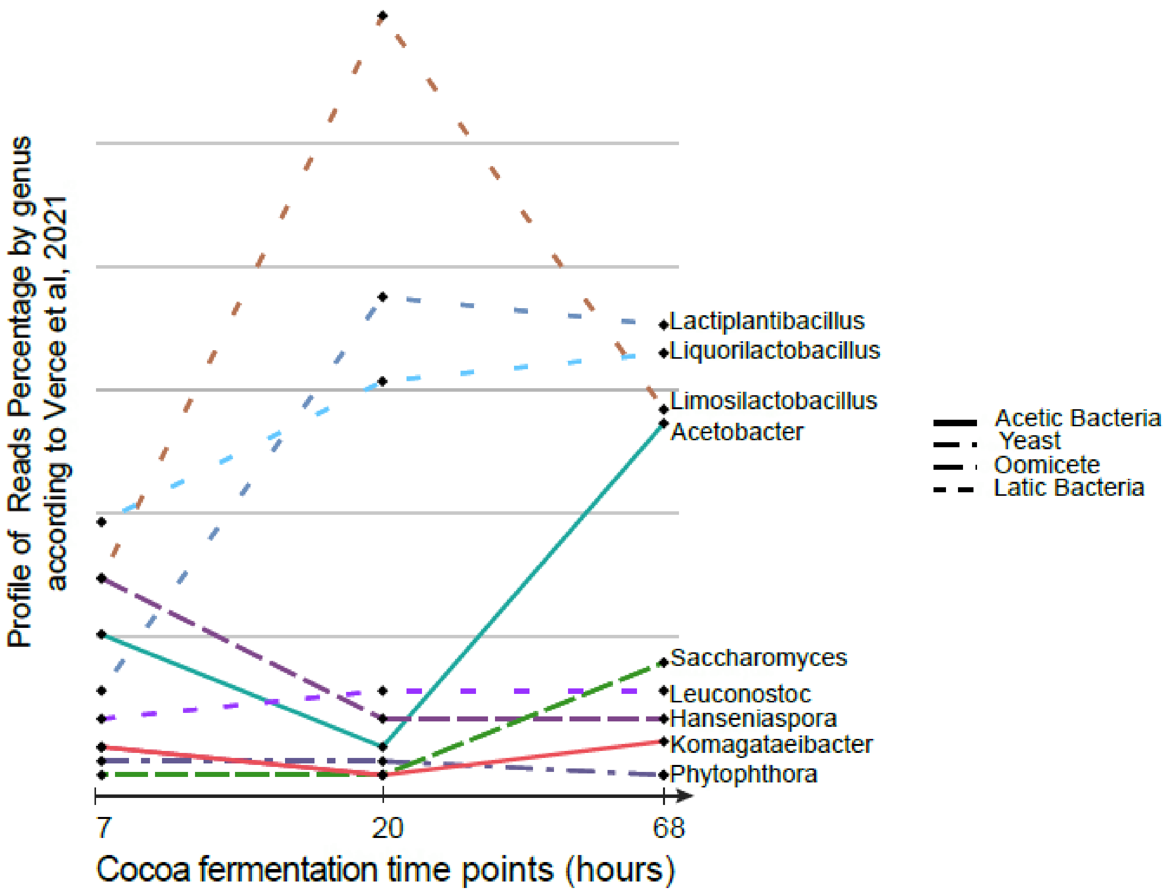

Figure S3. **The percentage profile of reads in each library, as outlined in the article by Verce et al. (2021).** The percentage profile of reads for the nine most abundant microorganisms throughout the cocoa fermentation process, with the general profile of the most abundant microorganisms based in the Supplementary Figure 1 from Verce et al. (2021). Each line pattern represents a specific microorganism group: acetic bacteria (solid line), yeast (large hatched line), oomycetes (dotted hatched line), and lactic bacteria (small hatched line).

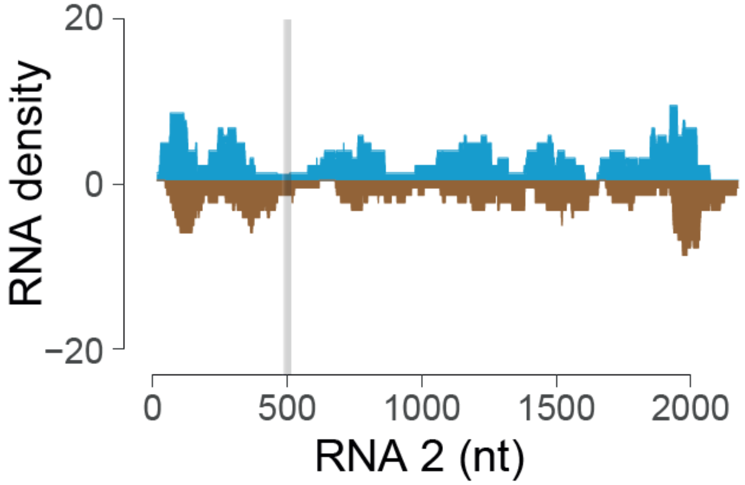

Figure S4. **Density of RNAs along the merged TcNV-5 RNA2 sequence.** The alignment location between the 3' end of RNA2.2 and the 5' end of RNA2.1 identified in gray. Positive sense strand is the depicted in blue and negative sense strand is depicted in brown.
